# Future of Triple Negative Breast Cancer: Can Immunotherapy Treat This Deadly Subtype of Breast Cancer?

**Published:** 2018-03

**Authors:** Mona Salimi

**Affiliations:** Department of Physiology and Pharmacology, Pasteur Institute of Iran, Tehran, Iran

## Triple negative breast cancer (TNBC): challenges and solutions via the immune cells

TNBC is one of the most complicated types of breast cancer to treat. It is generally diagnosed based on the absence of three receptors: estrogen, progesterone, and human epidermal growth factor receptor 2 (HER2) and is thus defined as a triple negative. TNBC is often more aggressive with lower survival rates than other forms of breast cancer; however, the outlook depends on the stage of diagnosis. This type of breast cancer cannot attract potent anti-cancer and hormone drugs due to the lack of surface proteins, so patients diagnosed with the disease have to rely almost exclusively on chemotherapy. In recent years, immunotherapy has gained attention, as a developing option, to treat a subset of cancers.

Previously, researchers have found a correlation between the presence of tumor-associated macrophages (TAMs) and poor prognosis in human cancers. However, a recent work has revealed that macrophages can be stimulated to phagocytose tumor cells, and this therapeutic plan has been exploited for multiple clinical trials in cancer. In addition to macrophages, other immune-regulatory receptors could also play a complementary role in immunotherapy of cancer. One of the clinically successful immune-check point drug targets, which is well studied, is the programmed death-1 (PD-1) receptor with a primary function in the regulation of T cells. In fact, targeting immune checkpoint proteins including cytotoxic T-lymphocyte antigen 4 and the PD-1 receptor revolutionized the treatment of some metastatic cancers. In this regard, it has been found that the inhibitory action of PD-1 bound to its ligand (PD-L1) suppresses the immune defense mechanism, which is exploited by the cancer cells. In this mechanism, up-regulation of cell surface PD-L1 expression in tumor cells allows the tumor cells to evade the immune detection, which subsequently leads to tumor progression. Additionally, recent studies have reported a high level of PD-1 expression in human TAMs, and that the stage of disease directly correlates with the level of PD-1 expression. Phagocytosis also appears to decrease due to PD-1 expression in TAMs; thus, PD-L1 removal may lead to PD-1 + TAM phagocytosis and a subsequent reduction in tumor burden, along with a functional recovery of TAMs. It is worth mentioning that PD-1 expression inhibits a wide variety of immune cell subsets, including T cells, B cells, natural killer cells, dendritic cells, and macrophages, in the tumor microenvironment, proposing that PD-1 expression is a global mechanism to restrict immunity, provided via the innate and adaptive immune system. Clinicians have exploited this mechanism for the benefit of breast cancer treatment.

Currently, the use of anti-PD-1/PD-L1 in treatment of breast cancer has received much attention, particularly for the TNBC subtype. TNBC tumors with a highly invasive characteristic express a large amount of PD-L1 and a high degree of tumor-infiltrating lymphocytes compared with other subtypes of breast cancer, implicating the immunogenic nature of TNBC.

## Current clinical trials testing for immunotherapy

Currently, an 18% overall response rate is achieved in the phase Ib clinical trial with the PD-1 antibody, pembrolizumab, for intensely-treated TNBC. In this trial, PD-L1 positivity was assigned to the cancer cells expressing PD-L1 by more than 1%, and anti PD-1 antibody response was correlated with the increasing expression of PDL-1. Moreover, a 33% general response was obtained for an anti-PD-L1 antibody, atezolizumab in a phase I trial for metastatic TNBC. An additional study performed on atezolizumab proposed that tumor response is associated with high levels of PDL-1 expression, not in tumor cells, but in tumor-infiltrating immune cells. Furthermore, the anti-PD-1 nivolumab (BMS-936558/MDX-1106) and the anti-PD-L1 durvalumab (MEDI4736) are presently under investigation in breast cancer.

Keeping in mind that the immune system consists of specific checkpoints vital for its function, manipulation of the effects of these natural checkpoints, using therapeutic antibodies would be promising to either control or eliminate tumors. Thus, hopefully, antibodies targeting and blocking immune checkpoints can change the standard of care for TNBC as a deadly subtype of breast cancer.

## More details in:

***Neoadjuvant Interferons: Critical for EffectivePD-1-Based Immunotherapy in TNBC***. NK Brockwell *et al*. 2017. Cancer Immun Res; Vol. 5, pp. 871-884.

***PD-1 expression by tumour-associated macrophages inhibits phagocytosis and tumour immunity***. SR Gordon *et al*. 2017. Nature; Vol. 545, pp. 495-499.

***Novel mechanism of immune evasion involving PD-L1 in various cancers*.** K Kaoka *et al*. 2016. Cancer Res; Vol. 5, pp. 428-S32.

***Programmed death ligand 1 expression in triple-negative breast cancer is associated with tumour-infiltrating lymphocytes and improved outcome***. RK Beckers *et al*. 2016. Histopathology; Vol. 69, pp. 25-34.

***Immunogenic subtypes of breast cancer delineated by gene classifiers of immune responsiveness***. LD Miller *et al*. 2016. Cancer Immunol Res; Vol. 4, pp. 600-610.

***Pembrolizumab in patients with advanced triple-negative breast cancer: phase Ib KEYNOTE-012 study***. R Nanda *et al*. 2016. *J Clin Oncol*; Vol. 34. pp. 2460-2467.

***PD-1/PD-L1 pathway in breast cancer*.** F Schütz *et al*. 2017. Oncol Res Treat; Vol. 40. pp. 294-297

